# Draft Genome Sequence of 16SrIII-J Phytoplasma, a Plant Pathogenic Bacterium with a Broad Spectrum of Hosts

**DOI:** 10.1128/genomeA.00602-16

**Published:** 2016-06-30

**Authors:** Alan Zamorano, Nicola Fiore

**Affiliations:** Universidad de Chile, Facultad de Ciencias Agronómicas, Departamento de Sanidad Vegetal, Santiago, Chile

## Abstract

Phytoplasmas are bacterial plant pathogens that can affect different vegetal hosts. In South America, a phytoplasma belonging to ribosomal subgroup 16SrIII-J has been reported in many crops. Here we report its genomic draft sequence, showing a total length of 687,253 bp and a G+C content of 27.72%.

## GENOME ANNOUNCEMENT

Phytoplasma diseases are widely distributed around the world and many of the species have been reported in South America (1, 2). Among them, particularly the 16SrIII-J ribosomal subgroup has been detected in many crops, from herbaceous to woody plants, mainly in Argentina, Brazil, and Chile (3–7). Two efficient insect vectors of 16SrIII-J phytoplasma were found in Chile and different weeds have been reported as reservoirs of the same phytoplasma (8–10). Therefore, knowledge of the genome of this phytoplasma is of great concern for the agronomy sanitary status in South America. DNA extract was obtained from single infected periwinkle plants according to a previous report (11). The extraction protocol was modified by an RNase A digestion step, before adding phenol-chloroform. Sequencing was done in two steps. First, DNA extracts from infected and healthy periwinkle were analyzed using Illumina paired-end sequencing. Phytoplasma reads were obtained as described (12). Sequences from infected periwinkle were *de novo* assembled using VELVET (13) and mapped against negative periwinkle reads, discarding all contigs with coverage lower than 10× and those that matched with one or more pair of reads, obtaining 475 contigs. All the reads contained in the contigs were separated and assembled using CELERA assembler (14), obtaining 176 scaffolds with a total length of 720,327 bp. In a second step, genome sequencing was continued using Ion Torrent PGM mate-pair sequencing using only infected periwinkle DNA extract. Total reads were trimmed by coverage lower than 10× and then were mapped against the total reads of the phytoplasma obtained in step 1. The final 101,204 reads were used for scaffolding, obtaining 29 scaffolds with a total length of 687,253 bp, *N*_50_ of 45,393, and a minimum and maximum size of 2,702 and 113,168 bp. G+C content reached 27.72%. Six hundred ninety-six coding sequences (CDSs), with 303 coding for hypothetical proteins, and 34 tRNAs, were identified using RAST, according to instructions (http://rast.nmpdr.org). The *IdpA* gene, coding for an immunodominant membrane protein specific for the X-disease group, was identified with 85% of nucleotide identity with Vaccinium witches’ broom phytoplasma, isolate VAC, and 85% of positives in amino acid sequence (76% of identity) with Italian clover phyllody phytoplasma, isolate MA1. Using BLASTx in web and local databases, 11 CDSs containing the SEC translocase complex signal peptide were identified but no SAP11, SAP54, PHYL, and TENGU homologous genes were found. To our knowledge, this is the first draft sequence of phytoplasma 16SrIII-J. Further studies are in progress to obtain the complete genome sequence.

### Nucleotide sequence accession numbers.

This whole-genome shotgun project has been deposited at DDBJ/EMBL/GenBank under the accession number LLKK00000000. The version described in this paper is version LLKK01000000.

## References

[1] Bertaccini A, Duduk B 2009 Phytoplasma and phytoplasma diseases: a review of recent research. Phytopathol Mediterr 48:355–378.

[2] Pérez-López E, Luna-Rodríguez M, Olivier CY, Dumonceaux TJ 2016 The underestimated diversity of phytoplasmas in Latin America. Int J Syst Evol Microbiol 66:492–513. doi:10.1099/ijsem.0.000726. 26519050

[3] Montano HG, Davis RE, Dally EL, Pimentel JP, Brioso PST 2000 Identification and phylogenetic analysis of a new phytoplasma from diseased chayote in Brazil. Plant Dis 84:429–436. doi:10.1094/PDIS.2000.84.4.429. 30841165

[4] Galdeano E, Torres LE, Meneguzzi N, Guzmán F, Gomez GG, Docampo DM, Conci LR 2004 Molecular characterization of 16S ribosomal DNA and phylogenetic analysis of two X-disease group phytoplasmas affecting China-tree (*Melia azedarach* L.) and garlic (*Allium sativum* L.) in Argentina. J Phytopathol 152:174–181. doi:10.1111/j.1439-0434.2004.00822.x.

[5] Gonzalez F, Zamorano A, Pino AM, Paltrinieri S, Bertaccini A, Fiore N 2011 Identification of phytoplasma belonging to X-disease group in cherry in Chile. Bull Insectology 64:S235–S236.

[6] Guzmán F, Giolitti F, Fernández F, Nome C, Lenardon S, Conci L 2014 Identification and molecular characterization of a phytoplasma associated with sunflower in Argentina. Eur J Plant Pathol 138:679–683. doi:10.1007/s10658-013-0352-y.

[7] Fiore N, Zamorano A, Pino AM 2015 Identification of phytoplasmas belonging to the ribosomal groups 16SrIII and 16SrV in Chilean grapevines. Phytopathogenic Mollicutes 5:32–36. doi:10.5958/2249-4677.2015.00060.2.

[8] Fiore N, Longone V, Gonzalez X, Zamorano A, Pino AM, Quiroga N, Picciau L, Alma A, Paltrinieri S, Contaldo N, Bertaccini A 2015 Transmission of 16SrIII-J phytoplasma by *Paratanus exitiosus* (beamer) leafhopper in grapevine. Phytopathogenic mollicutes 5:S43–S44. doi:10.5958/2249-4677.2015.00060.2.

[9] Quiroga N, González X, Zamorano A, Pino AM, Picciau L, Alma A, Paltrinieri S, Contaldo N, Bertaccini A, Fiore N 2015 Transmission of 16SrIII-J phytoplasma by *Bergallia valdiviana* Berg 1881 leafhopper. Phytopathog Mollicutes 5:S47–S48.

[10] Zamorano A, Gonzalez X, Pino AM, Quiroga N, Fiore N 2015 Detection of 16SrIII-J phytoplasma in *Galega officinalis* L., a weed commonly associated to pome fruit orchards in Chile. Phytopathog Mollicutes 5:S115–S116.

[11] Boudon-Padieu E, Béjat A, Clair D, Larrue J, Borgo M, Bertotto L, Angelini E 2003 Grapevine yellows: comparison of different procedures for DNA extraction and amplification with PCR for routine diagnosis of phytoplasmas in grapevine. Vitis 42:141–149.

[12] Saccardo F, Martini M, Palmano S, Ermacora P, Scortichini M, Loi N, Firrao G 2012 Genome drafts of four phytoplasma strains of the ribosomal group 16SrIII. Microbiology 158:2805–2814. doi:10.1099/mic.0.061432-0. 22936033

[13] Zerbino DR, Birney E 2008 Velvet: algorithms for de novo short read assembly using de Bruijn graphs. Genome Res 18:821–829. doi:10.1101/gr.074492.107. 18349386 PMC2336801

[14] Myers EW, Sutton GG, Delcher AL, Dew IM, Fasulo DP, Flanigan MJ, Kravitz SA, Mobarry CM, Reinert KH, Remington KA, Anson EL, Bolanos RA, Chou HH, Jordan CM, Halpern AL, Lonardi S, Beasley EM, Brandon RC, Chen L, Dunn PJ, Lai Z, Liang Y, Nusskern DR, Zhan M, Zhang Q, Zheng X, Rubin GM, Adams MD, Venter JC 2000 A whole-genome assembly of *Drosophila*. Science 287:2196–2204. doi:10.1126/science.287.5461.2196. 10731133

